# Risk factors of the antenatal depression in a sample of Italian pregnant women: a preliminary study

**DOI:** 10.1186/s12884-024-06704-8

**Published:** 2024-10-21

**Authors:** Maria Rita Sergi, Aristide Saggino, Michela Balsamo, Laura Picconi, Luigi Anchora, Marco Tommasi

**Affiliations:** 1 Department of Psychology, University of Chieti-Pescara, Chieti, Italy; 2 Family Counselling Centre, Maglie, Italy

**Keywords:** Antenatal depression, Assessment, Teate depression inventory, Pregnancy, Risk factors

## Abstract

**Background:**

Antenatal depression is characterized by low mood, insomnia, disorganised behaviour, irritability, and agitation during the pregnancy. If underestimated, antenatal depression is untreated during the pregnancy. It is associated to higher levels of suicide, higher risk of depression after childbirth, preeclampsia, preterm birth, low birth weight, poor interactions between child and mother and severe obstetric outcomes. New data underlined the importance to prevent the risk of depression during the pregnancy. This study examines the predictive validity of potential risk factors, such as socio-demographic and psychological factors, in developing the antenatal depression.

**Methods:**

The sample was composed by Italian pregnant women (*N* = 247, mean age of 33.77, SD = 4.78 years). This sample completed the Edinburg Postnatal Depression Scale (EPDS), the Teate Depression Inventory (TDI) and questionnaires about demographic variables. To study associations among variables examined bivariate correlations were computed. To analyse the role of socio-demographic factors and the psychological dimension to predict the severity of the antenatal depression a logistic regression was performed.

**Results:**

Results showed significantly positive correlations between the EPDS and the TDI, and no associations among the EPDS and all socio-demographic factors. Therefore, only the psychological factors were significant predictive risk factors of antenatal period. Finally, higher score of the depression measured via TDI predicted higher score of the EPDS.

**Conclusions:**

Our results had implications in clinical field. Indeed, the early diagnosis of depression during the pregnancy can help operators in the gynaecological field to prevent the depression in the post-partum period.

## Introduction

Antenatal depression (AD) is a specifier in depressive disorders as indicated “with peripartum onset” [1], characterized by low mood, insomnia, disorganised behaviour, irritability, and agitation during pregnancy.

Recent data showed that the risk to develop AD is underestimated, in relation to problems about instruments for its assessment because of their insufficient psychometric properties [2–5]. In particular, an untreated depression during the pregnancy is associated to higher levels of suicide, post-partum depression, preeclampsia, preterm births, low birth weight, poor interactions between child and mother and obstetric outcomes (such as the use of epidural analgesia and longer labour) [6–22].

Given the close association between post-partum depression and AD, it appears to be important to study risk factors (socio-demographic and psychological factors) during the pregnancy that can increase the level of AD. Psychological characteristics should be assessed through instruments with adequate psychometric properties that could predict precisely the risk of AD [16, 23–27].

Regards the impact of demographic variables on AD, a recent systematic review showed that the depressive effects of mothers’ age, mothers’ socioeconomic status or life events associated to stress (e.g.: a job loss) during the pregnancy is yet unclear [17, 28–30]. Indeed, some studies showed that divorce, separation or widowhood could predict the AD [16, 31–35]. Other researches, on the contrary, found no significant association between marital status and AD [36–43]. Regarding the occupational employment and educational level, there was a general consensus that unemployment and lower education levels were linked to AD [16, 31, 38, 44–52]. However, some studies reported opposite data. Al -Hejji et al. [53] and Chen et al. [54] found that higher levels of AD were associated to higher education levels. Pampaka et al. [49] also found a significantly positive association between AD and secondary education. However, other studies found no significant associations between AD, occupational employment and educational level [38, 39, 42, 55].

There are several studies that reported an association between age and AD. Andersson et al. [8] and Rubertsson et al. [15] found that younger women tended to have higher level of AD. Similar results were provided by Fellenzer and Cibula [31] in women between 18 and 24 years. In addition, younger age resulted to be a predictive factor of the AD especially during the second and the third trimester of pregnancy [56]. Also Rich-Edwards and colleagues [57] found that young pregnant women (≤ 23 yrs.) had a greater likelihood to develop AD than older ones. These results were confirmed by other studies [46, 58]. Recent studies, on the contrary, evidenced that an advanced maternal age (≥ 35 years) is more associated to the AD [59–63]. Additionally, no significant associations between AD and maternal age were found by other researchers [33, 36, 37, 39–44, 49, 52, 54, 64–67]. Recent meta-analyses and systematic reviews examine the risk factors for depression in pregnancy, including unemployment, marital status, social support, experience of violence, unplanned pregnancy, history of depression, smoking before pregnancy, and history of abortion [16, 20, 68]. It is important to analyze the risk factors for depression during pregnancy in order to prevent physical and psychological harm in the postpartum period for the woman and the child [20, 30].

AD is also linked to state psychological factors, in particular to depressive symptoms [69]. Recent studies showed as depressive symptoms were important predictive risk factors of post-partum depression [37, 70]. Up to date, only one study [71] concomitantly analysed the role of socio-demographic and state psychological factors on women’s depression during pregnancy. The research showed that only women’s age and depressive symptoms were the most important predictive risks factors of post-partum depression. Two systematic reviews [30, 72] highlight how the literature has focused on the postpartum period, neglecting the prenatal period for several reasons: the discrepancy between a decline in mood during pregnancy and social expectations of happiness can contribute to women’s greater difficulty in sharing feelings of unhappiness, sadness, and irritability; there is a belief that hormones protect women during pregnancy from psychological disorders; greater attention was devoted to the physical health of the woman and the fetus. In this psychophysiological context, there is a diagnostic difficulty between symptoms related to pregnancy (e.g., fatigue, complaints, depressed mood) and symptoms related to depressive symptomatology [11,22,72,]. One of the most widely used tools for antenatal depression is the Edinburgh Postnatal Depression Scale, which has poor ability to differentiate between depressed and non-depressed pregnant women. Moreover, the instrument has poor sensitivity to depressive symptoms, and poor discrimination power between somatic symptoms of depression and typical somatic symptoms of pregnancy, leading to false positives. The lack of psychometric properties can lead to un-diagnosed depression, with social and health burden [18, 20, 73]. For this reason, the present study employed the Teate Depression Inventory (TDI) [74–77], a new measurement tool for Major Depressive Episode, according to the criteria of the Diagnostic and Statistical Manual of Mental Disorders [1]. The instrument is constructed with Andrich’s Extended Logistic Model [78–80], which represents the broadest extension of the Rasch model for polytomous items, allowing discrimination between healthy subjects and pathological subjects. In particular, the TDI has good sensitivity and specificity, allowing for the discrimination of severity levels of depression through ROC Curves (Receiver Characteristic Curves).

The aim of our study is to analyse the predictive power of the principal demographic factors (age, marital status, occupational employment, educational level) and the risk of depression which showed to be relevant for predicting AD. We developed the following hypotheses:

1. H1: only demographic factors are significant predictive risk factors of AD.
2. H2: only the psychological factors are significant predictive risk factors of AD.
3. H3: both demographic and psychological factors are significant predictive risk factors of AD. In particular, we conducted a comparison between demographic and psychological factors to study which of these weigh more on the risk of depression.

## Materials and methods

### Participants

247 women who attended antenatal courses participated in this study. Their mean age was 33.77 years (SD = 4.78). Regards the marital status 80 women (32.4%) were single (never married, separated, divorced, widowed) and 167 (67.6%) were married or cohabiting. As the occupational employment, 47 women (19%) were unemployed and 200 (81%) were employed. As the educational level, 178 women (72.1%) had completed high school, and 69 (27.9%) had a university degree. 29 subjects (11.7%) had a past miscarriage; 16 subjects (6.5%) had a past voluntary abortion, and 2 subjects (0.8%) underwent voluntary termination of pregnancy by decision of others. Moreover, 84 pregnant women (34.0%) experienced adverse events in life and high perceived stress, and 34 subjects (13.8%) had personal history of mental illness such as anxiety, relationship problems, or mourning. Finally, 21 (8.5%) pregnant women drank before pregnancy, and 8 (3.2%) subjects drank only with meals during pregnancy. Additionally, 83 (33.6%) pregnant women smoked before pregnancy, and 10 (4.0%) subjects smoked during pregnancy. The sample was in the third trimester of pregnancy, during which there is a higher prevalence of depression [21, 30, 72].

### Measures

**Measures of demographic characteristics.** Questions related to demographic characteristics were provided in the questionnaire. The questions were about age (number of years), marital status, occupational employment, and educational level.

**Edinburg Postnatal Depression Scale** (EPDS) [81] is a 10-items self-report screening measure for antenatal and postpartum depression. Items have a Likert scale from 0 (no symptom) to 3 (severe symptom). The cut-off indicating a severe postnatal depression ranges from 6/7, in countries as Ethiopia and Greek [50, 82] to 12/13, in countries as Italy, Iran, Island, Portugal and Taiwan [82–86]. In this study a cut-off of 13 was used to identify the presence of severe symptoms of AD. In the Italian context the sensitivity of the scale is equal to 56%, the specificity is equal to 98% and the positive predictive value is equal to 83% [82, 86].

**Teate Depression Inventory** (TDI) [74] is a 21-item unidimensional self-report instrument of depression. The TDI is based on the DSM-5 diagnostic criteria for Major Depression Episode [1]. The frequency of each depressive symptom was measured with a Likert scale from 0 (never) to 4 (always). On the basis of the global score, individuals were categorized into four categories related to the severity of the depressive syndrome: minimal (0–21), mild (22–36), moderate (37–50) and severe depression (51–80). The external validity of this scale is evidenced by several cross-cultural studies [87–90].

### Procedures

Pregnant women were recruited at the Family Counseling Center, Italy, during antenatal courses. All measures were presented to pregnant women during specific days dedicated to risk factors of depression during the pregnancy. In these meetings the importance to measure the depression in the pregnancy as predictor of post-partum depression was explained. All instruments were administered by psychologists from July 2018 to May 2019. The Family Counseling Center’s aim is guarantee the reproductive health of women, promoting responsible motherhood and paternity, protecting childhood and primary prevention. The procedures of research followed in the present work are in accordance with the ethical standards of the institutional and national research committee and with the 1964 Helsinki declaration. Anonymity and privacy of the participants were guaranteed according the Italian and the European laws about privacy (Italian law n. 196/2003 and EU GDPR 679/2016, respectively). Informed signed consent was obtained from all individual participants. Participation was voluntary. The study was ethically approved by the Family Counseling Center (Protocol Number = 116682).

### Statistical analyses

Multivariate test of normality was made on empirical data (Mardia’s test). In addition, the internal consistency of the psychological scales was estimated through Cronbach’s alpha [91] and McDonald’s omega [92].

Bivariate correlations were computed among the TDI, the EPDS scores and the demographic variables. Marital status (unmarried or married), occupational employment (employed or unemployed) and educational level were transformed into variables with dummy coding. When variables were dichotomous we used the dummy values “0” (unmarried, unemployed) and “1” (married, employed). Educational levels were transformed into an ordinal variable with values from 0 (high school) to 1 (university degree). To analyse the predictive validity of socio-demographic and psychological factors on the EPDS total score we performed a linear regression. A hierarchical analysis was performed with two models: in the first model EPDS scores were predicted only by demographic factors, while in the second model the TDI score was added as a psychological predictor.

To analyse the AD predictive risk validity of socio-demographic psychological factors we performed a logistic regression. In the logistic regression all demographic variables and the TDI total score were used as predictors of severe symptoms of AD (EPDS cut off score ≥ 13). Statistical analyses were calculated using the Statistical Package for the Social Sciences (SPSS 25) [93] and JASP Version 0.11.1.0 [94].

## Results

### Descriptive statistics

Means, standard deviations, skewness, kurtosis and reliability of the two scales are shown in Table 1. Skewness and kurtosis showed values between in the range ± 1, supporting a normal distribution of these data [95]. The obtained McDonald’s omega and Cronbach’s alpha for the TDI indicated an excellent scale reliability; while the EPDS indicated a discrete scale reliability [96].

**Table 1:** Mean, standard deviation, normality indices and internal consistency of the TDI and EPDS (*N* = 247)

	Mean	SD	Skewness	Kurtosis	McDonald’s omega	Cronbach’s alpha
TDI	14.17	9.299	1.024	1.324	0.910	0.905
EPDS	5.35	3.812	0.749	0.210	0.785	0.76

### Depression and demographic characteristics

Table 2 showed the bivariate correlations between the EPDS total score and the TDI total score, as well as the demographic characteristics (age, marital status, educational level, and occupational employment). Results showed high significatively positive correlation between the EPDS and the TDI (*r* = .647; *p* < .05); while there was no significant correlation (*p* < .05) between the EPDS and all the examined demographic characteristics.

**Table 2:** Correlation among the EPDS total score with the TDI total score, age, marital status, educational level and occupational employment (*N* = 247)

	TDI	Age	Marital status	Educational level	Occupational employment
EPDS	0.647**	0.038	− 0.082	− 0.089	− 0.053

**p < .05

### Predictors of the EPDS score

Table 3 shows the results of the linear regression analysis. In the first model all the demographic variables (age, marital status, educational level and occupational employment) were the predictors of the EPDS total score. No predictor was significant. In the second model the TDI total score was added as predictor of the EPDS. Results showed that the TDI explained the 43% of variance of the EPDS (R Squared Change = 0.42), while all the demographic factors explained about the 2%.

**Table 3:** Linear regression analysis (*N* = 247)

Model 1	EPDS					
	β	B	A	t	P	sr^2^
Age	0.070	0.56	22.21	1.068	0.287	0.00
Marital status	-0.086	-0.700	-10.12	-1.330	0.185	0.00
Educational level	-0.104	-0.884	-10.336	-1.609	-0.109	0.01
Occupational employment	-0.044	-0.428	-10.26	-0.686	0.493	0.00
R²			0.021			
AR²			0.005			
F			1.315			
p (F)			0.265			

Model 2	EPDS					
	β	B	A	t	P	sr^2^
TDI	0.667	0.274	2.40	13.366***	0.000	0.42
Age	-0.048	-0.038	22.30	-0.959	0.338	0.00
Marital status	-0.106	-0.862	-9.96	-2.156*	0.032	0.01
Educational level	-0.042	-0.357	-10.86	-0.850	0.396	0.00
Occupational employment	-080	0.772	-11.46	1.600	0.111	0.00
R²			0.438			
AR²			0.426			
F			37.555			
p (F)			0.000			

Note. R² = R Square; AR²= Adjusted R Square; sr = Partial correlations. ***p < .001; *p < .05

### Predictors of the antenatal depression

Table 4 shows the results of the logistic regression used for predicting the antenatal depression, measured on the basis of the EPDS (≥ 13). Only the TDI total score was the best predictive risk factor of AD (EPDS ≥ 13). In particular, higher the score of the TDI, higher the score of the EPDS (B=. 170; Wald = 20.282; *p* < .001).

**Table 4:** Logistic regression for predicting the antenatal depression (*N* = 247)

	EPDS				
Age	B	S.E.	Wald	*p*	Odd ratio (95% CI)
Age	0.061	0.07	0.799	0.371	1.063 (.930-1.214)
Marital status	-1.3	0.68	3.661	0.056	0.274 (.073-1.032)
Educational level	0.133	0.77	0.03	0.862	1.142 (.255-5.116)
Occupational employment	0.336	0.81	0.173	0.677	1.399 (.288-6.798)
TDI	0.17	0.04	20.282	< .001	1.185 (1.101–1.276)

## Discussion

Recent literature suggests that the AD is predictive index of the risk to develop post-partum depression or to suffer from severe birth outcomes (babies with extreme low weight at birth, preterm birthings, spontaneous abortion). The AD is associated with low self-esteem, low maternal responsiveness, high perfectionism and higher levels of anger [23, 65, 97–100]. These conditions can influence the children’s psychological development (e.g.: development of high insecurity attachment and of internalizing or externalizing disorders), probably because of an increase of maternal cortisol. Hypothetically, an exposure to a maternal distress leads to an increased cortisol, which is associated to an altered hypothalamic-pituitary-adrenal (HPA) function.

This alteration is linked to negative effects to the foetus [10, 101, 102]. For this reason, an accurate identification of the risk factors that generate AD can be helpful in developing a more precise early diagnosis of post-partum depression or risky pregnancy [24, 103].

Our research examined the predictive validity of potential risk factors in developing AD in a sample of Italian pregnant women. The examined risk factors were socio-demographic and state psychological factors, in particular the depressive symptomatology. Our result confirmed that only the psychological factors can be considered as significant predictive risk factors of AD. Our study showed that depressive symptoms developed during the pregnancy is the most significant predictive risk factor of depression in the antenatal period, in accordance with the previous studies [69–71, 104, 105]. Our study showed that demographic factors had no relevant predictive importance and, therefore, they could not be used as valid index to predict and prevent the risk to develop antenatal depression or to suffer from problematic pregnancy, in contrast with several previous studies [8, 15, 16, 31–35, 38, 44–52, 56, 57, 59–62]. These results were confirmed by previous studies focusing on socio-demographic risk factors [37–44, 49, 52, 54, 55, 67].

These results can have implications in the clinical treatment of problematic pregnancies. Our data pointed out the importance to make an accurate screening for the presence of depressive symptoms during pregnancy through valid and reliable instruments for psychological assessment. An early recognition of the AD allows the implementation of a clinical preventive psychological intervention to reduce the risk of post-partum depression and its fatal consequences [106]. Recent studies and several meta-analyses showed the efficacy of the cognitive and behavioural therapy (CBT) approach in treating and preventing the AD [107]. Indeed, in the CBT protocol for the antenatal depression, it is important to analyse the cognitive schemas regard representations of the parenting abilities and representations of children [108, 109]. In particular, the CBT focuses on cognitive restructuring of irrational beliefs and cognitive distortions, as well as the modification of dysfunctional behaviour [110–115]. Finally, our study uses a new instrument for measuring depression: the TDI, which has a greater discriminative capacity between depressed and non-depressed subjects compared to the self-report instruments for the diagnosis of depression most used in clinical field [74–77].

Our study had several limitations: the small number of the participants and the specificity of their geographic origin (southern part of Italy) may reduce the generalizability of our results. Further research is needed with a greater number of individuals recruited in other areas of the country, as well other variables associated with pregnancy. Furthermore, our measures were administered only during the pregnancy. We planned to perform longitudinal studies to analyze the risk factors of postpartum depression in the future. In particular, future research will focus on retesting the variables examined at 3–6 and 9 months of pregnancy. Finally, the study primarily focused on the psychological state of pregnancy. Future studies will also investigate other risk factors, such as lack of partner or of social support, history of abuse or of domestic violence abortion, adverse events in life and high perceived stress, and personal history of mental illness, although they are characteristics that may be completely absent or present in very few pregnant women.

In conclusion, the results of our study evidenced that the psychological traits of women during the pregnancy, more than their demographic characteristics, are the relevant elements to consider in preventing the risk of depression during the pregnancy [116]. In addition, an early diagnosis of depression during the pregnancy can help operators in the gynaecological field to detect and prevent the risk of depression in the post-partum period [117].

## Data Availability

The data that support the findings of this study are available on request from the corresponding author. The data are not publicly available due to privacy or ethical restrictions.
